# Effects of a Rosiridin against Rotenone-induced Rats Model of Parkinson's Disease: *In-vivo* Study and *in silico* Molecular Modeling

**DOI:** 10.2174/011570159X349553250126050134

**Published:** 2025-02-13

**Authors:** Misbahuddin Rafeeq, Fahad A. Al-Abbasi, Muhammad Afzal, Khalid Saad Alharbi, Ehssan Moglad, Salwa D. Al-Qahtani, Hussam A. Bukhari, Faisal Imam, Nadeem Sayyed, Imran Kazmi

**Affiliations:** 1 Department of Pharmacology, Faculty of Medicine, Rabigh King Abdulaziz University, Jeddah, Saudi Arabia;; 2 Department of Biochemistry, Faculty of Sciences, King Abdulaziz University, Jeddah 21589, Saudi Arabia;; 3 Department of Pharmaceutical Sciences, Pharmacy Program, Batterjee Medical College, P.O. Box 6231 Jeddah 21442, Saudi Arabia;; 4 Department of Pharmacology and Toxicology, College of Pharmacy, Qassim University, Qassim 51452 Saudi Arabia ;; 5 Department of Pharmaceutics, College of Pharmacy, Prince Sattam Bin Abdulaziz University, Alkharj 11942, Saudi Arabia;; 6 Department of Medical Laboratory Sciences, College of Applied Medical Sciences, Majmaah University, Al Majmaah 11952, Saudi Arabia;; 7 Department of Pathology, Faculty of Medicine, King Abdulaziz University, Jeddah 21589, Saudi Arabia;; 8 King Abdulaziz University Hospital, King Abdulaziz University, Jeddah 21589, Saudi Arabia;; 9 Department of Pharmacology and Toxicology, College of Pharmacy, King Saud University, P.O. Box 2457, Riyadh 11451, Saudi Arabia;; 10 School of Pharmacy, Glocal University, Saharanpur 247121, India

**Keywords:** Antioxidants, neurotransmitters, neuroprotective, oxidative stress, Parkinson's disease, rotenone

## Abstract

**Aim:**

The investigation aimed to study the outcome of rosiridin in Parkinson's disease (PD) induced by rotenone (ROT) in rodents.

**Methods:**

Rodents were randomized into IV groups and were induced with ROT followed by treatment with rosiridin. Group I-IV received saline as a vehicle, II-ROT (0.5 mg/kg S.C) for 28 consecutive days, III and IV- rosiridin 10 and 20 mg/kg orally with ROT. On completion of the experimental duration, behavioral investigations were carried out. Biochemical variables such as acetylcholinesterase (AChE), oxidative stress and antioxidants markers (Malondialdehyde-MDA, glutathione-GSH, superoxide dismutase-SOD, and catalase-CAT), anti-inflammatory (Interleukin-1 beta-IL-1β, IL-6, and tumor necrosis factor alpha-TNF-α), alteration in neurotransmitters (Serotonin-5-HT), norepinephrine, and dopamine-DA, along with metabolites such as 5-hydroxy indole acetic acid (5-HIAA), mitochondrial complex I, II, IV, and caspase-3 activity were evaluated at the end of the experiment. Furthermore, molecular docking and dynamics were performed for target ligands.

**Results:**

Rosiridin significantly restored the level of AChE, oxidative stress and antioxidants markers (MDA, GSH, SOD, and CAT), anti-inflammatory (IL-1β, IL-6, and TNF-α), alteration in neurotransmitters, mitochondrial complex I, II, IV, and caspase-3 activity. Rosiridin has a favorable negative binding affinity to AChE (-8.99 kcal/mol). The results of the molecular dynamics simulations indicate that proteins undergo a substantial change in conformational dynamics when binding to rosiridin.

**Conclusion:**

In this study, rosiridin may exhibit neuroprotective properties against the Parkinson's model for treating PD.

## 1 INTRODUCTION

Parkinson's disease (PD) is a neurodegenerative disorder characterized by motor symptoms such as bradykinesia, tremor, rigidity, and gait disturbances. It is also associated with a diverse range of non-motor complications, including cognitive, behavioral, and autonomic dysfunction [1-4]. PD prevalence of 1-2 per 1000 individuals, increases with age and affects 1% of those over the age of 60 years. 5-10% have genetic risk, which is more common in men than women [5]. The pathogenesis of PD is characterized by the progressive loss of dopaminergic neurons in the substantia nigra, leading to a deficit in dopamine neurotransmission. Oxidative stress, exacerbated by monoamine oxidase B-mediated dopamine degradation and glutamate accumulation, contributes to neuronal death [6]. The complete treatment of PD has not been identified, but therapeutic approaches are available to lower the symptoms, such as physical therapy and exercises. Pharmacological management of PD often involves a multi-drug approach that includes levodopa, COMT inhibitors (entacapone, Tolcapone), anticholinergics agents (Biperidine, Procyclidine), dopaminergic agonists (Bromocriptine, Ropinirole), and inhibitors of MAO-B (rasagiline or selegiline) to alleviate symptoms [7].

Rotenone (ROT) is a widely studied neurotoxin and is used to induce symptoms related to PD by inhibiting the mitochondrial complex I among experimental animals [8]. The mitochondrial complex is responsible for energy formation in the form of ATP [9, 10]. Studies identify that ROT results in the degeneration of selective neurons [11, 12]. This degeneration leads to the lowering of DA in the brain, which is the hallmark feature of PD. ROT is used for the identification of the mechanisms of PD and its potential treatments [13-15]. Extensive research has revealed that the neurotoxicity induced by ROT administration involves various factors such as inflammation, mitochondria dysfunctionality, oxidative stress, and impaired protein degradation [16, 17]. Consequently, these outcomes develop innovative therapeutic approaches that target these pathways to treat and prevent PD [18, 19].

Rosiridin is a flavonoid found in *Rhodiola rosea*. It has potential therapeutic properties, including anti-inflammatory, antioxidant, anti-cancer, and anti-depressant actions [20, 21]. However, no research has been conducted that supports its use in treating or preventing PD [22]. Antioxidants protect the cells from damage caused by free radicals. Few studies have proposed that extracts from *Rhodiola rosea* containing rosiridin, potentially have effects of neuroprotection and can be useful in the treatment of PD [23, 24]. Due to antioxidant properties, rosiridin neutralizes free radicals, thereby protecting cells from oxidative damage, and rosiridin is postulated to exert neuroprotective effects [22, 25].

However, this research aimed to assess the effect of rosiridin on PD among the rodents that were induced by ROT. Both experimental and computational methods, such as dynamic simulations and molecular docking were used to identify the target protein.

## 2 METHODS

### 2.1 Chemicals

ROT was received from Sigma Aldrich (USA). The rosiridin was obtained as a sample from MSW Pharma, India. ELISA kit carried out the Interleukins- (IL-1β), IL-6, TNF-α, and Caspase 3 analysis (Krishgen Biosystems, M.S. India).

### 2.2 Animals

Male Wistar rats weighing 180 ± 20 g and aged 10-12 weeks, were kept in cages made of propylene, and access to a pellet diet and tap water was provided. They were maintained under the light/dark cycle of 12 hours at a suitable temperature (23°C) and humidity level (50-65%). The behavioral parameter analysis commenced in the rats' active phase, between 19.00 and 24.00 hr. This study obtained permission from the IAEC for animals and was carried out by the ARRIVE guidelines. Animal obtaining details-LNCP, M.P., India and sample size- 24 rats (n=6).

### 2.3 Prediction of ADMET by Computational Analysis

The pharmacokinetic (PK) properties of rosiridin, including absorption, distribution, metabolism, excretion, and toxicity (ADMET), were assessed using pkCSM [26].

### 2.4 Experimental Groups

All the rats were grouped into four (n=6)


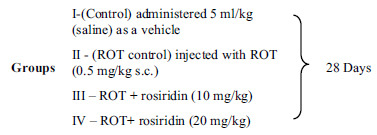


The dose of Rosiridin administered to rats was determined based on previous research and preliminary investigations [27]. Behavioral quantification of the rats was conducted on the 29^th^ day, followed by the biochemical analysis.

### 2.5 Body Weight

The body's weight was measured on the first day before the ROT injection and the last day.

### 2.6 Behavioral Parameters

#### 2.6.1 Catalepsy Test

The catalepsy test assesses the motor coordination and stability among the rats. It involved the placement of a rat on a bar of wooden with its hind limbs on a raised surface and its forepaws to be placed on the floor. The time the rat took to correct its posture and maintain stability on the elevated surface was marked. The repetition of this test was carried out six times to obtain the mean value as the measurement of catalepsy [28].

### 2.7 Rotarod Test

The activity of rotator was carried out to evaluate motor skills and grip control among the animals. Rats were subjected to a pre-training phase to identify the test duration. Each rat kept on a rotating rod (diameter - 7 cm) was supposed to be rotated at 25 rpm. The time taken to fall from the rod was recorded, with a maximum limit of 180 seconds to overcome the stress [29].

### 2.8 Open Field Test

Plexiglass and wood apparatus were used to identify natural motor behavior in rats. Nine central squares and sixteen outer squares crossed by the rats were recorded, and behaviors like licking the paw and fur also observed. Sniffing, standing upright on hind limbs, and leaning against the wall using forelimbs were observed. This test assessed the natural motor activity and associated changes in the behavior of the rats [30, 31].

### 2.9 Biochemical Assessment

The brain of the rat was collected on the last day of the experiment with utmost care and homogenized with a solution of phosphate buffer at 0.1 M concentration. This homogenate was centrifugated at a speed ranging from 15,000 rpm to 20,000 rpm for 20 minutes. The resulting supernatant was carefully collected and subjected to subsequent biochemical analysis.

### 2.10 Acetylcholinesterase (AChE) Activity

The AChE activity assay kit is a direct procedure for measuring AChE levels. Spectrophotometric measurements of samples of brain tissue homogenate were taken at 410 nm. The action of AchE was quantified as µmol/g, showing enzyme-related activity among the samples [32-34].

### 2.11 Oxidative and Antioxidant Markers

Oxidative stress markers like MDA, GSH, SOD, and CAT were estimated. The MDA levels indicate cellular damage due to lipid peroxidation, while the CAT enzyme reduces oxidative stress by breaking down hydrogen peroxide (H_2_O_2_) the amount of MDA, GSH, SOD and CAT was determined using the previously reported method [35].

### 2.12 Neurotransmitter Levels

Serotonin (5-HT), norepinephrine, and dopamine (DA), along with metabolites such as 5-hydroxy indole acetic acid (5-HIAA), were measured using HPLC [36-40].

### 2.13 Neuroinflammatory Markers

For the quantification of IL-1β, IL-6, and TNF-α, ELISA kits were used [41-43].

### 2.14 Estimation of Mitochondrial Complex I Activity

The standard procedure extracted the mitochondria complex I activity from rat brains. The brain was homogenized and spectrophotometrically measured at 37°C for 3 min by NADH oxidation at the wavelength of 340 nm inside the assay medium [44, 45].

### 2.15 Estimation of Mitochondrial Complex II Activity

The activity of mitochondrial complex II was measured by observing the gradual lowering of nitro blue tetrazolium (NBT) to a product with an insoluble color in nature called formazan (pdf), which was further detected at 570 nanometers wavelength. The initiation of this particular reaction was performed by introducing a mitochondrial preparation to the mixture of reaction composed of 1.5 ml phosphate buffer, 0.3 ml BSA, 0.2 ml succinic acid (0.6 M, pH 7.8), and 0.1 ml 0.03 M potassium ferricyanide [44, 45].

### 2.16 Estimation of Mitochondrial Complex IV Activity

The cytochrome-C oxidase identification was conducted using the Storrie and Madden method, which was given in 1990. The procedure involved reducing cytochrome c by adding a few sodium borohydride crystals and then achieving a neutral pH using 0.1 M HCl. The reaction progressed by introducing mitochondrial suspension in 0.075 M phosphate buffer and adding the reduced cytochrome c. The reduction in absorbance at 550 nm was monitored for 3 minutes [45].

### 2.17 Caspase-3 Estimation

The Caspase-3 concentrations were determined using an ELISA kit. The results were expressed as pg/mL to indicate the concentration of Caspase-3 in the samples.

### 2.18 Histopathology

Rats were euthanized and decapitated, followed by the immediate removal and fixation of brain tissue in 10% buffered formalin. After dehydration through a graded ethanol series, the tissues were embedded in paraffin. Using a light microscope, a histopathological examination was conducted on 5 µm thick paraffin sections stained with hematoxylin and eosin (H&E).

### 2.19 Molecular Docking

For the docking study, three-dimensional structures of five proteins (IL-6 (PDB ID: 1ALU), TNF α (PDB ID: ZAZ5), AChE (PDB ID: 7XN1), Casp-3 (PDB ID: 1NME) and DA (PDB ID: 6CM4)) were obtained from the RCSB protein database. Protein structural optimization and minimization were performed using CHIMERA v1.16 2, incorporating force fields for standard (AMBER ff14SB) and nonstandard residues (AM1-BCC). Consequently, all nonstandard residues like water molecules & cocrystal ligands, and unnecessary chains have been removed from the proteins. We got the ligand rosiridin from ChemSpider and cleaned it in MarvinSketch underwent 2D and 3D cleaning. Cleaned Structures then subjected to MMFF94 force field for minimization, and the lowest energy conformer was selected for further study in MOL2 format. We used AutoDockTools 1.5.6, Chimera 1.11, and Maestro Version 12.7.161 were employed for grid Generation and validation. Grid Parameters were obtained using orientation of co-crystal ligand or CASTp^6^ server if protein in Apo state. We used the co-crystal ligand or CASTp server to get the grid parameters. We converted the ligand and proteins to pdbqt format using Bash scripts. We performed docking with AutoDock Vina, setting the grid box at the active site of each protein. We used AutoDock Vina 1.2.57,8 for docking studies, with 0.375 Å of spacing between the grid points. The grid box was centered on the active site of the target. grid parameters like Centre & size Coordinates were followed as IL-6 1ALU (9.966, -20.835, 16.696 and 35, 35, 35) TNF-α ZAZ5 (-19.41, 74.65, 33.85 and 25, 25, 25), AChE 7XN1 (48.32, -40.32, -30.0 and 40, 40, 40), Caspase-3 1NME (42.09, 96.34, 24.13 and 25, 25, 25), and Dopamine 6CM4 (9.93, 5.85, -9.58 and 25, 25, 25) and other parameters like CPU were set for 23, exhaustiveness was 32, number of modes were 9 and energy range was set for 3. We used Biovia Discovery Studio, LigPlot, and Maestro to visualize the protein-ligand complexes in 2D and 3D. We used a PLIP server to find the interactions between them. We reported the details in Table **1**.

### 2.20 Molecular Dynamics Simulation

The simulations of molecular dynamics studies were done on the D2 DA Receptor (6CM4) and human acetylcholinesterase (7XN1) in Apo state (6CM4_Apo and 7XN1_Apo) and dock complex with rosiridin (6CM4_Rosiridin & 7XN1_Rosiridin) by the use of Desmond 2020.1 from Schrödinger, LLC. Samplings - triplicate using similar parameters for each run to procure the reproducible findings (Table **2**). OPLS-1005 force field [46, 47] and an explicit model of the solvent with SPC molecules of water were used [48]. The addition of sodium ions was conducted to neutralize the charge. The NaCl solutions of 0.15 M were added to stimulate the system's physiological environment. The equilibrium was performed using an NVT ensemble for 100 ps. It was set up using the Nose-Hoover technique followed by short-run equilibrium [49] with 27ºC, 2.0 ps, and at 2 bar. Martyna-Tuckerman-Klein [50] method for controlling pressure with the relaxation duration - 2 ps. The particle mesh Ewald method was used for the calculation of wide-range interactions, and the coulomb interaction radius was fixed at 9Å. The RESPA integrator was used to calculate the bonded forces. For monitoring the stability of MD simulations, RMSD, RMSF, Rg, Hydrogen Bonds, and radius of Gyration (Rg) were calculated.

### 2.21 Binding Free Energy Analysis

The molecular mechanics in combination with generalized Born surface area (MM-GBSA) for computing binding free energies of 6CM4_Rosiridin and 7XN1_Rosiridin. The MM-GBSA method was employed to compute the binding free energy (ΔGbind) between a ligand and its receptor. The calculation utilized the thermal_mmgbsa.py Python script, analyzing the final 50 frames of a molecular dynamic’s simulation conducted with the OPLS_2005 force field. A 100-step sampling approach was implemented within each frame to enhance the precision. ΔGbind was derived using the principle of additivity, where individual energy components, including Columbia (electrostatic), hydrogen bonding, covalent, van der Waals, self-contact, solvation, lipophilic, and pi-stacking interactions, for both the ligand and receptor, were summed. The specific equation used to calculate ΔG_bind_ is provided below:


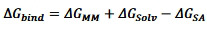


### 2.22 Statistical Examination

The obtained outcomes were expressed as mean ± SEM. Numerical variables were tested using Shapiro-Wilk tests. One-way ANOVA followed by Tukey's Post hoc test was used to compare various variables within each group and assess the significance of the differences using Graph Pad Prism software (Version 8.0.2). In the context of statistical analysis, the results were considered statistically remarkable if *P <* 0.05.

## 3 RESULTS

### 3.1 ADMET Properties

ADMET (pkCSM) was used to evaluate the pharmacokinetic properties of rosiridin, as presented in Table **3**.

### 3.2 Behavioral Parameters

#### 3.2.1 Catalepsy Test

Fig. (**1A**) shows motor activities in groups. The ROT injection depicted a remarkable elevation in the catalepsy duration compared to the control group. Rosiridin treated in both groups significantly lowered catalepsy time compared to the ROT-injected group (F (3, 20) = 66.59, (*p* < 0.0001)).

#### 3.2.2 Rotarod Test

**Fig. (1):**
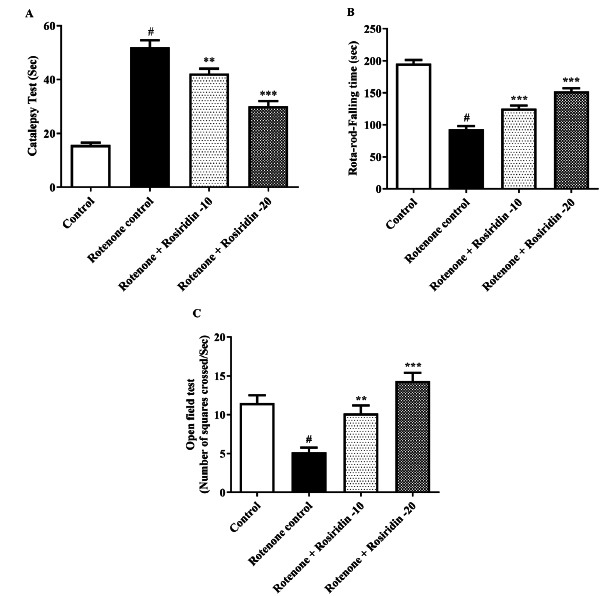
(**A-C**) Effects of rosiridin on behavioral test: (**A**) Catalepsy, (**B**) Rotarod, and (**C**) Open Field test. The findings in the present study were quantified by mean ± S.E.M (*n* = 6). A one-way ANOVA followed by Tukeys *post hoc*, **p < 0.001, ***p < 0.0001 *vs*. ^#^*p*< 0.05 rotenone control group.

Rats with PD induced by ROT exhibited significant motor impairments, as demonstrated by a decrease in the time spent on the rotarod and an increase in the number of falls compared to a control group. Conversely, rats treated with both doses of rosiridin significantly improved their performance on the rotarod compared to ROT-induced PD rats (F (3, 20) = 67.28, (*p* < 0.0001)) and showed a marked reduction in motor deficits (Fig. **1B**).

#### 3.2.3 Open Field Test

The number of squares was notably lower in ROT injected groups than in the control. Treatment with rosiridin in both doses showed a significant increase in the traveled distance as compared to ROT-injected rats (F (3, 20) = 16.87, (*P <* 0.0001)) (Fig. **1C**).

### 3.3 Biochemical Estimation

#### 3.3.1 Acetylcholinesterase Activity

**Fig. (2):**
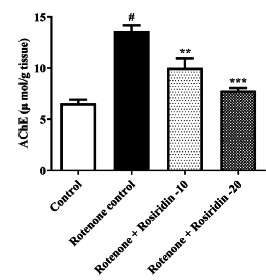
Effects of rosiridin on acetylcholinesterase (AChE). The findings in the present study were quantified by mean ± S.E.M (*n* = 6). A one-way ANOVA followed by Tukeys *post hoc*, **p < 0.001, ***p < 0.0001 *vs.* ^#^*p* < 0.05 rotenone control group.

The ROT injected group exhibited a rise in AChE level when correlated to the control group. Lower and higher dose groups of rosiridin resulted in significantly reduced AChE activity among the ROT-injected rats (F (3, 20) = 28.70,(*P <* 0.0001)) (Fig. **2**).

### 3.4 Oxidative Stress Markers

**Fig. (3):**
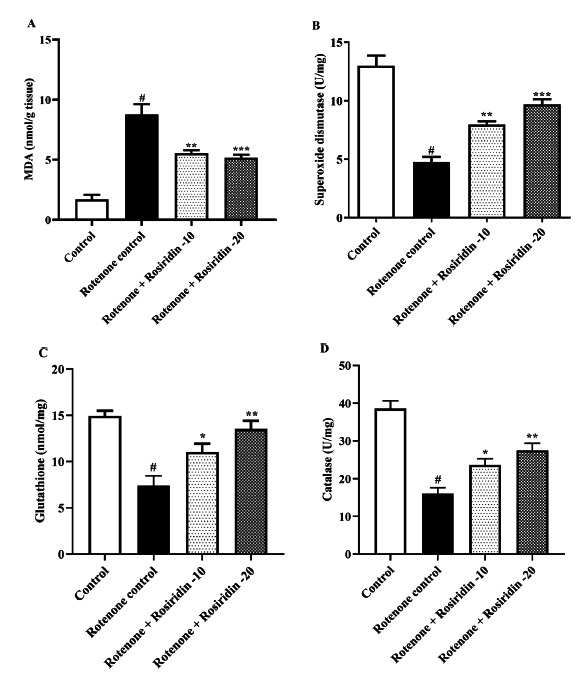
(**A-D**). Effects of rosiridin on oxidative stress and antioxidants markers (**A**) Malondialdehyde (MDA), (**B**) Superoxide dismutase (SOD), (**C**) Glutathione (GSH), and (**D**) Catalase (CAT). The findings in the present study were quantified by mean ± S.E.M (*n* = 6). A one-way ANOVA followed by Tukeys *post hoc*, *p < 0.05, **p < 0.001, ***p < 0.0001 *vs.* ^#^*p* < 0.05 rotenone control group.

Compared with control, MDA levels were raised (*P <* 0.0001) in ROT-injected rats. Rosiridin significantly declined MDA (F (3, 20) = 32.79, (*P <* 0.0001)) compared to ROT-injected rats. The GSH, SOD, and CAT levels were significantly lowered in ROT-injected rats when correlated with controls. The GSH (F (3, 20) = 14.12, (*P <* 0.0001)), SOD (F (3, 20) = 36.76, (*P <* 0.0001)), and CAT level (F (3, 20) = 27.49, (*P <* 0.0001)) on treatment with both doses of rosiridin were elevated considerably in comparison with ROT-injected rats (Figs. **3A**-**D**).

### 3.5 Neurotransmitter Levels

**Fig. (4):**
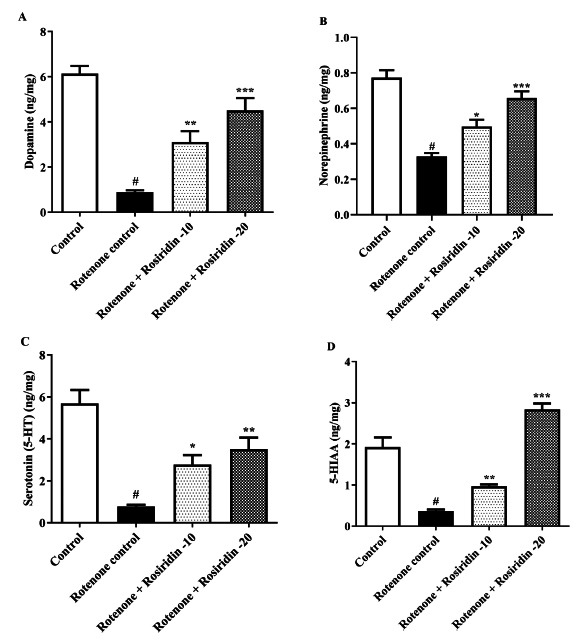
(**A-D**). Outcome of rosiridin on neurotransmitters (**A**) Dopamine (DA), (**B**) Norepinephrine, (**C**) Serotonin (5-HT), and (**D**) 5-hydroxy indole acetic acid (5 HIAA). The findings in the present study were quantified by mean ± S.E.M (*n* = 6). A one-way ANOVA followed by Tukeys *post hoc*, *p < 0.05, **p < 0.001, ***p < 0.0001 *vs.* ^#^*p* < 0.05 rotenone control group.

The DA, epinephrine, 5-HT, and 5-HIAA have decreased in the ROT-injected rats compared to the control group. Both doses of rosiridin significantly restored DA (F (3, 20) = 17.93, (*P <* 0.0001)), norepinephrine (F (3, 20) = 29.63, (*P <* 0.0001), 5-HT (F (3, 20) = 59.49, (*P <* 0.0001)), and 5-HIAA levels (F (3, 20) = 58.74, (*P <* 0.0001)) compared to ROT-injected rats (Figs. **4A**-**D**).

### 3.6 Neuroinflammatory Markers

**Fig. (5):**
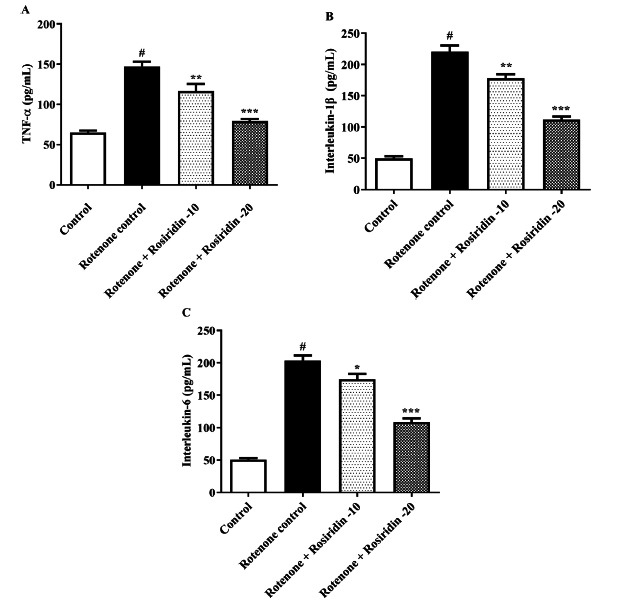
(**A-C**). Effect of rosiridin on (**A**) Tumor necrosis factor alpha (TNF-α), (**B**) Interleukin-1 beta (IL-1β), and (**C**) IL-6. The findings in the present study were quantified by mean ± S.E.M (*n* = 6). A one-way ANOVA followed by Tukeys *post hoc*, *p < 0.05, **p < 0.001, ***p < 0.0001 *vs.* ^#^*p* < 0.05 rotenone control group.

The ROT group exhibited increased proinflammatory markers levels compared to the control group. Treatment with both doses of rosiridin significantly reduced IL-1ß (F (3, 20) = 126.4, (*P <* 0.0001)), IL-6 (F (3, 20) = 115.8, (*P <* 0.0001)), and TNF-α (F (3, 20) = 45.20, (*P <* 0.0001)) compared to ROT-injected rats (Figs. **5A**-**C**).

### 3.7 Mitochondrial Complex-I, II, and IV

**Fig. (6):**
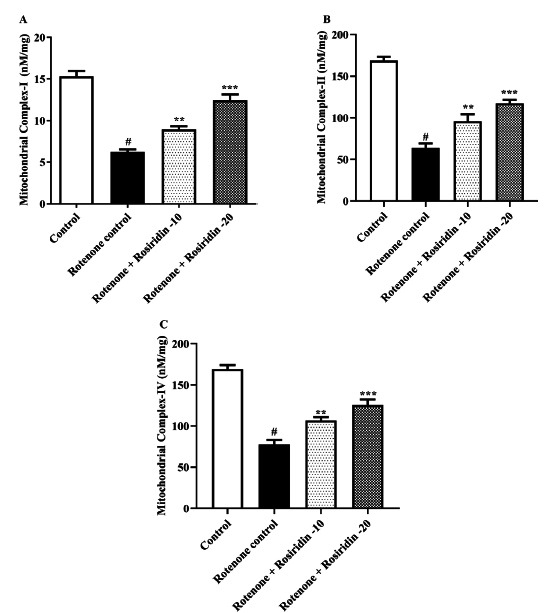
(**A-C**). Outcome of rosiridin on (**A**) Mitochondrial complex I, (**B**) Mitochondrial complex II, and (**C**) Mitochondrial complex IV. The findings in the present study were quantified by mean ± S.E.M (*n* = 6). A one-way ANOVA followed by Tukey’s *post hoc*, **p < 0.001, ***p < 0.0001 *vs.* ^#^*p* < 0.05 rotenone control group.

One-way ANOVA depicted the remarkable differences in NADH dehydrogenase (*P <* 0.0001), succinate dehydrogenase (*P <* 0.0001), and Cytochrome-C Oxidase (*P <* 0.0001) activity among the treated group. One-way ANOVA followed by Tukey's test showed that both doses of rosiridin significantly increased the mitochondrial complex-I (F (3, 20) = 54.47, (*P <* 0.0001)), mitochondrial complex-II (F (3, 20) = 57.03, (*P <* 0.0001)), and mitochondrial complex-IV (F (3, 20) = 72.90, (*P <* 0.0001)) ROT-injected decline in rat respiratory enzyme actions (Figs. **6A**-**C**).

### 3.8 Caspase-3 Estimation

**Fig. (7):**
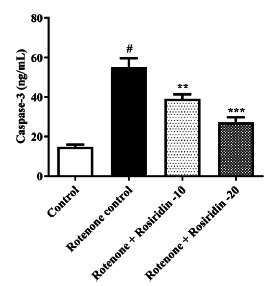
Effect of rosiridin on caspase 3. The findings in the present study were quantified by mean ± S.E.M (*n* = 6). A one-way ANOVA followed by Tukey`s *post hoc*, **p < 0.001, ***p < 0.0001 *vs.* ^#^*p* < 0.05 rotenone control group.

The study results revealed that ROT-injected rats exhibited upregulation of caspase-3 compared to the control group. One-way ANOVA followed by Tukey's test showed that both doses of rosiridin led to a significant downregulation in caspase-3 expression (F (3, 20) = 34.39, (*P <* 0.0001)) compared to ROT-injected rats (Fig. **7**).

### 3.9 Histopathology

The protective properties of rosiridin on brain tissue, as evaluated through hematoxylin and eosin (H&E) staining, are depicted in Figs. (**8A**-**D**). ROT-induced PD led to significant neuronal degeneration. Rosiridin, at a dose of 10 mg/kg, attenuated the deleterious effects of ROT, evident in the reduced number of deeply stained neurons. The improved brain histology demonstrated a more pronounced protective effect with rosiridin at a dose of 20 mg/kg.

### 3.10 Molecular Docking

**Fig. (9):**
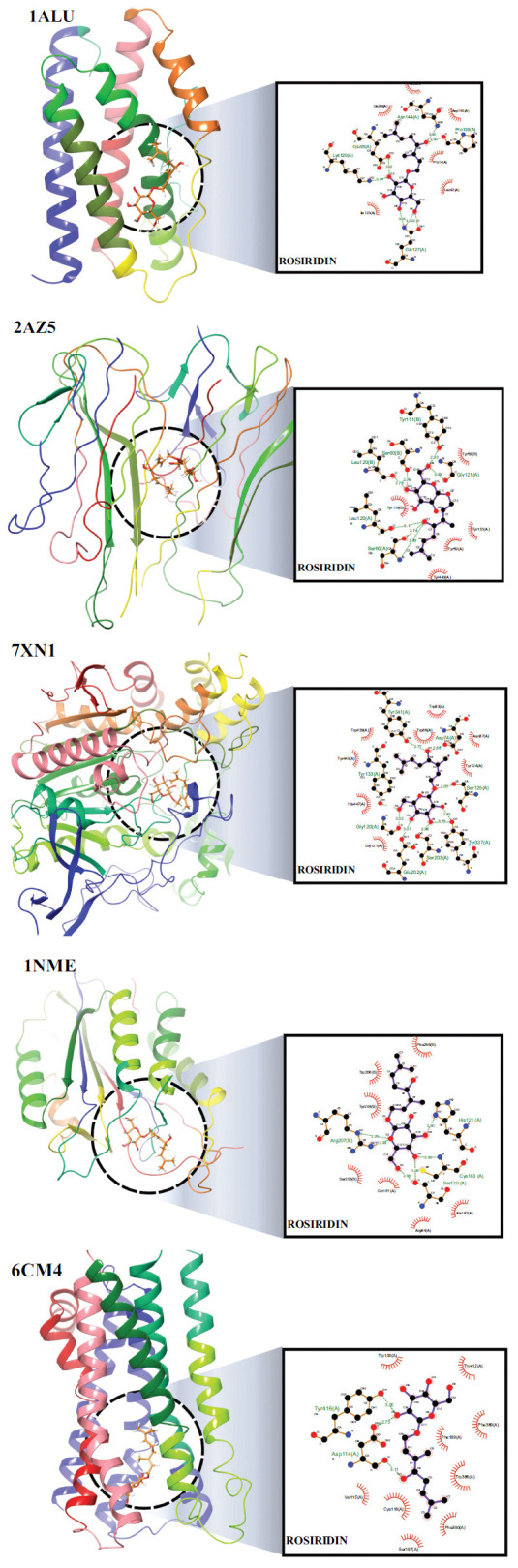
Molecular docking 2D and 3D images of the Rosiridin with against proteins IL-6 (PDB ID: 1ALU), TNF α (PDB ID: ZAZ5), AChE (PDB ID: 7XN1), Caspase-3 (PDB ID: 1NME) and Dopamine (PDB ID: 6CM4) using LigPlot v1.4.5 Maestro V12.8 software.

Based on the docking results (Table **2**), rosiridin has a favorable binding affinity to AChE (-8.99 kcal/mol), followed by Dopamine (-7.36 kcal/mol), Caspase-3 (-6.62 kcal/mol), TNF-α (-6.39 kcal/mol), and IL-6 (-5.71 kcal/mol). These results show that rosiridin exhibits binding interactions with several proteins involved in PD, such asIL-6, TNF-α, AChE, caspase-3, and dopamine. Rosiridin may have potential neuroprotective effects by inhibiting AChE activity associated with PD progression (Fig. **9**).

### 3.11 MD Simulation

**Fig. (10A-D):**
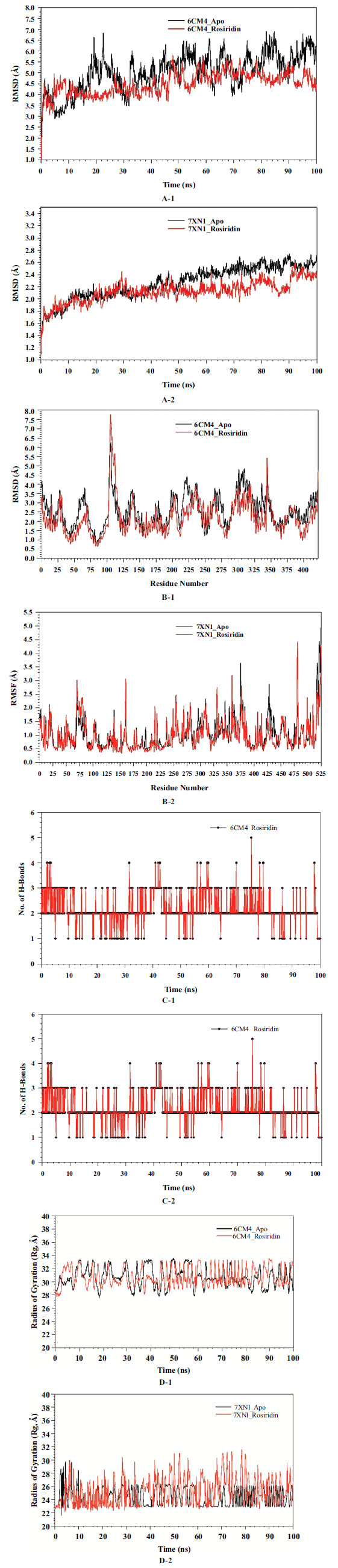
Analysis of MD simulation trajectories of 100 ns time scale. (**A-1**) RMSD plot displaying the molecular vibration of Cα backbone of 6CM4_Apo form (Black) & 6CM4_Rosiridin (Red), (**A-2**) RMSD plot displaying the molecular vibration of Cα backbone of 7XN1_Apo form (Black) & 7XN1_Rosiridin (Red). (**B-1**) RMSF plots show the fluctuations of respective amino acids throughout the simulation time 100 ns for 6CM4_Apo form (Black) & and 6CM4_Rosiridin (Red). (**B-2**) RMSF plots show the fluctuations of respective amino acids throughout the simulation time 100 ns for 7XN1_Apo form (Black) & 7XN1_Rosiridin (Red). (**C-1**) A number of hydrogen bonds formed in 6CM4_Rosiridin (Red) during a 100 ns simulation time scale. (**C-2**) A number of hydrogen bonds formed in 7XN1_Rosiridin (Red) during a 100 ns simulation time scale. (**D-1**) The radius of gyration plots for the deduction of compactness of 6CM4_Apo form (Black) & 6CM4_Rosiridin (Red). (**D-2**) The radius of gyration plots for the deduction of compactness of 7XN1_Apo form (Black) & 7XN1_Rosiridin (Red).

The convergence and stability of both Apo and rosiridin-bound forms of 6CM4 and 7XN1 were studied using MD and simulation to evaluate conformational stability. A comparison of RMSD of the simulation of the trajectories of 100 ns showed stable conformation. RMSD of the Cα-backbone of the unbound form of proteins, 6CM4_Apo and 7XN1_Apo, was 5.03 Å & 2.26 Å, respectively, on average (Figs. **10 A1**-**A2**) while in the bound state, 6CM4_Rosiridin & 7XN1_Rosiridin was found to be 4.50 Å & 2.12 Å respectively. 6CM4_Apo shows substantial fluctuations in RMSD, with values ranging roughly between 3.5 Å to over 6.5 Å. This indicates significant conformational changes as the protein moves and adapts over time, while the protein with a Rosiridin-bound state of both proteins shows lower RMSD, ranging between 3.0 Å to just under 5.5 Å for 6CM4 and 1.35 Å to 2.57 Å for 7XN1, which might be stabilizing the protein, reducing the extent of conformational changes compared to the Apo form.

The RMSD trends suggest that both complexes achieved equilibrium after an initial period. Rosiridin appears to have induced a slightly higher degree of structural deviation in 6CM4 compared to its Apo form, whereas 7XN1 displayed a more consistent RMSD profile in both Apo and bound states. RMSF measured the flexibility of the Cα-backbone of 6CM4 & 7XN1 in the bound and unbound states (Figs. **10 B1**-**B2**). The pattern of sharp peaks and valleys reflects protein regions that are more or less flexible. Troughs that are consistent across both forms suggest structural rigidity that is maintained irrespective of ligand binding. The rest of the residues conformed to α-Helices and β-sheets. In the 6CM4 RMSF graph, it has been found that a peak is present in the Apo form, which is reduced in the ligand-bound form, noticeable around residue numbers 50, 175, and 350, which suggests that ligand binding may be stabilizing that region of the protein. The RMSF graph of 7XN1 shows specific areas of the protein experienced increased flexibility when bound to rosiridin, as evidenced by the higher RMSF values in the rosiridin-bound state. The presence of rosiridin induces higher fluctuations in some regions, suggesting increased local flexibility, which might be necessary for the protein's functional activity. Conversely, 7XN1 shows a different pattern, with some areas exhibiting decreased fluctuations upon rosiridin binding, which could indicate the stabilization effects of the ligand. The H-bonds numbers between ligand and protein indicate the remarkable stability and interaction of the complex. Remarkable H-bonds were observed. The hydrogen bonds formed between 6CM4_Rosiridin (Average 2.16) and 7XN1_Rosiridin (Average 2.57) complexes displayed temporal fluctuations, indicating dynamic interaction landscapes (Figs. **10 C1**-**C2**). Notably, on average, the 7XN1_rosiridin complex demonstrated a higher number of hydrogen bonds, suggesting a potentially more robust or stable interaction with the ligand than the 6CM4_Rosiridin complex. This indicates that rosiridin interacts dynamically with proteins 6CM4 and 7XN1, attributed to the specific binding site geometry or differences in the electrostatic and hydrophobic interactions facilitated by the residues involved.

The Rg analysis for the protein-ligand complexes 6CM4 and 7XN1 over a 100 ns molecular dynamics simulation revealed how ligand binding influences the compactness of these proteins (Figs. **10 D1**-**D2**). For 6CM4, the Rg values fluctuate between 28 and 36 Å, with the Apo and Rosiridin-bound states showing similar trends. In contrast, the 7XN1 complex exhibits a tighter Rg range, particularly in the rosiridin-bound state, suggesting a more compact and less variable structure upon ligand binding. The Rg values for 6CM4 show that the protein maintains its compactness on binding with ligand binding without changes in the tertiary structure suggesting that the binding site of eosiridin in 6CM4 is pre-formed that allows ligand accommodation without major conformational changes. The slight increase in the variability of the Rg in the bound form around 60 ns indicate the transient conformational changes or flexibility that is important for the functioning of protein and release of ligands. The 7XN1 protein showed a decline in structural variability when bound to rosiridin. This decrease implies a stabilizing effect of the ligand on the structure of a protein that increases the binding affinity and specificity due to a reduction in entropic costs during the binding process. Comparison of the Rg profiles of 6CM4 and 7XN1 suggested that the two proteins respond differently to rosiridin binding at the structural level. 6CM4 retains a similar degree of compactness and variability upon ligand binding and 7XN1 adopts a more stable and compact structure. For 6CM4, targeting the transiently flexible states might enhance ligand binding, whereas, for 7XN1, ligands that capitalize on the reduced structural variability could be more effective.

### 3.12 Post-simulation Binding Free Energy Analysis

The MM-GBS method, a widely used computational technique, was employed to estimate ΔG_bind_ of 6CM4_rosiridin and 7XN1_rosiridin complexes with their respective protein targets. Additionally, the contributions of various interaction energies were evaluated. Table **4** shows the ΔG_bind_ of 6CM4_rosiridin and 7XN1_rosiridin revealed by the dynamics.

The average binding energies of the ligand in 6CM4 displayed -46.71±4.46 kcal/mol, while in 7XN1, -29.22±5.03 kcal/mol (Table **4**). Interestingly, ΔG_bind SolvGB_ and ΔG_bind Covalent_ exhibited energy contributions that were not favorable (positive values), indicating their opposition to binding. Generally, more negative ΔGbind values indicate stronger binding affinity, as observed for 6CM4_Rosiridin and 7XN1_Rosiridin.

## 4 DISCUSSION

Rosiridin effectively mitigates the behavioral and biochemical abnormalities induced by ROT in rats. Moreover, rosiridin was observed to alleviate the toxic impact of ROT on the dopaminergic system in rats [51]. At a sub-cellular level, rosiridin counteracted the adverse consequences of ROT by improving the antioxidant actions of enzymes and preserving function and bioenergetics. These findings underscore the potential of rosiridin as a promising alternative for managing PD and emphasize the therapeutic advantages associated with using this natural compound [52, 53].

According to the pkCSM ADMET prediction server, a reliable tool for toxicity assessment, rosiridin exhibits moderate to low intestinal absorption, with a rate of 41.83%. Furthermore, its BBB penetration was estimated to be less than one [54]. The administration of rosiridin resulted in a discernible amelioration of the behavioral changes induced by ROT among the rats [48]. A deficit in the DA level is a key factor perturbing normal motor functions, leading to clinical indications such as tremors, muscle rigidity, and impaired control in PD [55].

In this study, exposure to ROT resulted in a progressive diminishment in locomotor capabilities and a concomitant increase in cataleptic manifestations. These specific behavioral indices are extensively recognized as pivotal determinants that impact PD. The unity of these findings is in line with the antecedent research [56]. The primary contributor to the pathogenesis of PD is prominently linked to the formation of ROS due to oxidative damage. Mitochondrial dysfunction is a salient contributor to ROS formation, adversely affecting cellular moieties such as lipids, DNA, and proteins. This damage further exacerbates the pathogenic processes associated with PD [57]. Pro-survival pathways activated by vitagenes mediate the cellular stress response. These protective genes orchestrate the synthesis of antioxidant and antiapoptotic molecules, such as heat shock proteins, glutathione, and bilirubin. Vitagenes, critical for aging, stress, and protein homeostasis, ensure the efficacy of cellular stress responses and protein quality control. By regulating antioxidant production, vitagenes mitigate oxidative stress, a pivotal factor in neurodegenerative diseases like PD [58-60].

As revealed in prior studies, a robust correlation exists between ROT-induced motor impairments and the oxidative stress state within the ROT disrupts ATP production, leading to an imbalance in the production and detoxification of ROS. Similarly, in the present study, ROT elevated the MDA level and a concomitant diminished level of GSH (endogenous antioxidant), SOD, and CAT [61, 62]. This accumulation of ROS triggers oxidative stress, damaging cellular components and compromising neuronal function. Rosiridin exerted protective effects against ROT-induced oxidative stress by modulating the levels of oxidative stress markers. It reduced the levels of MDA, suggesting a synergistic effect in scavenging ROS and preventing lipid peroxidation. Administration of rosiridin increased the levels of other parameters, leading to a more robust antioxidant defense system and detoxification of superoxide radicals, contributing to the efficient conversion of hydrogen peroxide and reducing oxidative stress.

The study demonstrated that ROT-elevated brain IL-1β, IL-6, and TNF-α can reduce cerebral blood flow. These effects were significantly mitigated by the administration of rosiridin, suggesting that rosiridin may exert its neuroprotective effects by suppressing the neuroinflammatory response triggered by ROT [63].

The results showed that the systemic injection of rats with ROT reduced the AChE activity. This leads to the accumulation of acetylcholine in the brain and potentially impacts cognitive function and muscle control by disrupting cholinergic signaling pathways. The lowered AChE activity develops tremors, rigidity, and bradykinesia. Treatment with rosiridin dose-dependently reduced AChE activity, suggesting neuroprotective effects by preserving the cholinergic function and potentially alleviating cognitive deficits. In line with previous research, ROT in this study markedly declines mitochondrial function [55-57]. In the present study, rosiridin increases the activity of mitochondrial complexes (I, II, and IV), suggesting that rosiridin can directly counteract the inhibitory effects of ROT and restore normal function.

Our current study demonstrates a significant reduction in dopamine in ROT-administered rats, consistent with findings from prior investigations [64]. ROT administration decreased DA, neither epinephrine, 5-HT, and 5-HIAA catecholamine levels [62, 65]. DA and 5-HT deficiency and other alterations within the striatal neurochemistry balance have been found to occur as PD progresses [66]. The reduction in DA, nor-epinephrine, 5-HT, and 5-HIAA levels caused by ROT was increased in the rat by administration of rosiridin. The effects of rosiridin on the dopaminergic system have been documented by other animal models of PD. These findings suggest that rosiridin possesses the ability to potentiate dopaminergic activity, potentially mediated by alterations in the activity of striatal dopaminergic system metabolic enzymes, ultimately leading to an improvement in motor function in the ROT-induced rats [45, 67].

Administration of ROT exhibits a detrimental effect on neurons by increasing the activity of caspase-3. This increase triggers a cascade of events that ultimately leads to the destruction of neurons. It indicated that rosiridin administration significantly decreases the activity of caspase-3, thereby protecting neuronal function by reducing apoptosis and promoting cell survival in PD.

Histopathological analysis revealed that rosiridin, at both doses, mitigated PD induced by rotenone. The current findings demonstrate that rosiridin preserved neuronal architecture and reduced neuronal degeneration, resulting in normal brain histology. These results align with previously reported studies [68].

The docking results of rosiridin had a favorable binding affinity to AChE (−8.99 kcal/mol), followed by Dopamine (−7.36 kcal/mol), Caspase-3 (−6.62 kcal/mol), TNF-α (−6.39 kcal/mol), and IL-6 (−5.71 kcal/mol). This indicates that rosiridin has a neuroprotective effect by blocking AChE activity associated with PD progression [69-71]. The MD simulation results indicate that rosiridin exhibits distinct mechanisms of action on proteins 6CM4 and 7XN1, which may influence their roles in neurotransmission. Rosiridin stabilizes and enhances 6CM4, facilitating increased dopamine transport across the synaptic cleft. The interaction with 6CM4 promotes a more stable protein conformation, as evidenced by lower root mean square deviation (RMSD) values over the simulation period. In contrast, rosiridin acts as a modulator and inducer of 7XN1, affecting acetylcholine hydrolysis and ligand binding. The compound exerts differential effects on the conformational stability and flexibility of 6CM4 and 7XN1. Notably, contributions from solvation-free energy and covalent interactions could have been more substantial in determining the final binding energy values. This study revealed that ROT-treated rats exhibit a robust oxidative stress response, profound mitochondrial dysfunction, catecholamine depletion, and elevated apoptosis, highlighting potential therapeutic targets. Consequently, attenuating excessive free radical generation, augmenting antioxidant defenses, preventing neurochemical deficiencies, or modulating inflammatory pathways may hold promise in mitigating the emergence of ROT-induced behavioral deficits [72-74]. Notably, rosiridin demonstrates neurotransmitter-protective and mitochondrial-rescuing properties alongside potent anti-oxidant inflammatory actions, suggesting its potential therapeutic efficacy based on our findings [74]. Rosiridin inhibits casp-3 and reduces the accumulation of toxic dopamine metabolites. It helps maintain the DA levels in the brain and minimizes the motor symptoms of PD. Further investigation, encompassing quantitative immunohistochemical analyses and positive control to strengthen the findings and provide a more comprehensive comparison, is needed to validate the observed effects of rosiridin on neurotransmitter levels in the context of PD and establish its therapeutic potential. Limitations of the current study include the relatively short duration of the experiment and the use of a smaller sample size.

## CONCLUSION

This study demonstrates that rosiridin exhibits significant neuroprotective effects in a rotenone-induced PD model by restoring antioxidant balance, reducing neuroinflammation, modulating neurotransmitter levels, and preserving mitochondrial function while also inhibiting apoptosis (caspase-3). AChE binding studies confirmed rosiridin has a strong affinity for AChE (-8.99 kcal/mol), suggesting its potential role in cholinergic regulation. As a result of these findings, rosiridin appears to be a promising candidate for treating PD by targeting oxidative stress, neuroinflammation, mitochondrial dysfunction, and neurotransmitter imbalances. This study provides preclinical evidence that rosiridin might be a promising treatment for Parkinson's disease. Further studies, including dose optimization and long-term efficacy studies, are required to validate these effects.

## Figures and Tables

**Fig. (8):**
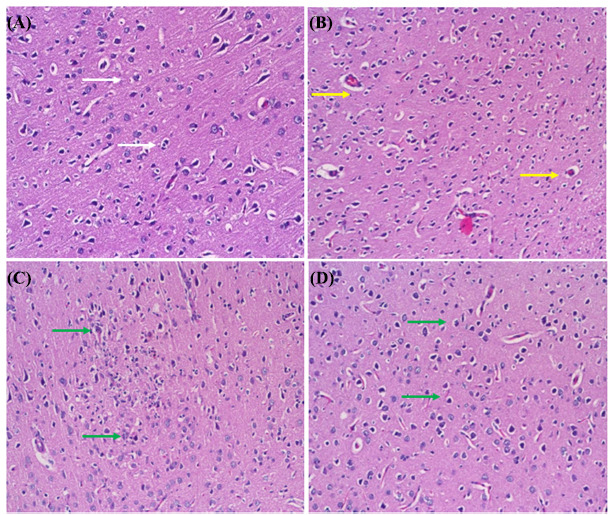
(**A-D**). Histopathological changes stained with hematoxylin and eosin X200 in the midbrain of rats: (**A**) Control group showed normal histoarchitecture (White arrow), (**B**) ROT control group decreased neuron size and neuronal damage (Yellow arrow). (**C** and **D**) Rats treated with rotenone and rosiridin 10 and 20 mg showed a good degree of protection of the substantial neurons (Green arrow).

**Table 1:** Docking score and intermolecular interactions of ligand (Rosiridin) against proteins IL-6 (PDB ID: 1ALU), TNF-α (PDB ID: ZAZ5), AChE (PDB ID: 7XN1), Caspase-3 (PDB ID: 1NME) and Dopamine (PDB ID: 6CM4).

**Sr. No.**	**Name of Complex**	**Binding Energy (Kcal/Mol)**	**Type of Interaction**	**Residue Id**	**Distance In Å**
1	1ALU	-5.706	Hydrophobic Interactions	GLU95A	3.82
				PRO139A	3.7
			Hydrogen Bonds	GLU95A	2.43
				LYS120A	2.12
				GLN127A	2.17
				PRO139A	1.93
				ASN144A	2.62
			Salt Bridges	LYS120A	5.26
2	2AZ5	-6.389	Hydrophobic Interactions	TYR59A	3.71
				TYR119A	3.7
				TYR119A	3.49
				TYR119A	3.43
			Hydrogen Bonds	SER60A	2.35
				SER60B	3.13
				SER60B	2.22
3	7XN1	-8.994	Hydrophobic Interactions	ASP74A	3.42
				TRP86A	3.47
				TRP86A	3.85
				TYR337A	3.71
				TYR337A	3.5
				TYR449A	3.38
			Hydrogen Bonds	ASP74A	3.36
				GLY120A	2.4
				TYR124A	3.02
				SER125A	2.32
				TYR133A	2.67
				SER203A	2.24
				TYR337A	2.34
				TYR341A	1.96
				HIS447A	2.75
4	1NME	-6.615	Hydrophobic Interactions	PHE256B	3.7
				PHE256B	3.65
			Hydrogen Bonds	ARG64A	3.05
				ARG64A	2.47
				SER120A	2.08
				HIS121A	1.81
				GLY122A	3.12
				GLN161A	2.15
				CYS163A	2.08
				TYR204B	3.15
			Salt Bridges	ARG207B	3.46
5	6CM4	-7.36	Hydrophobic Interactions	PHE189A	3.85
				PHE198A	3.98
				TRP386A	3.85
				TRP386A	3.7
				PHE389A	3.68
				PHE390A	3.66
			Hydrogen Bonds	THR412A	2.71
				TYR416A	2.36

**Table 2:** Molecular dynamics parameters.

**Parameter**	**Details**
Software	Desmond 2020.1 (Schrödinger LLC)
Force field	OPLS-2005
Ensembles	NVT (100 ps), NPT (1 ns)
Time step	2 fs /100ns
Temperature	27ºC
Pressure	2 bar
Solvent model	SPC water model
Ion concentration	0.15 M NaCl
Charge neutralization	Na^+^ ions
Electrostatics method	Particle Mesh Ewald
Pressure control	Martyna-Tuckerman-Klein barostat with 2 ps relaxation

**Table 3:** Predicted ADMET properties of rosiridin.

**Property**	**Model Name**	**Predicted Value**	**Unit**
Absorption	Water solubility	-1.971	Numeric (log mol/L)
	CaCo_2_ permeability	-0.132	Numeric (log Papp in 10^-6^ cm/s)
	Intestinal absorption (human)	41.835	Numeric (% Absorbed)
	Skin Permeability	-2.99	Numeric (log Kp)
	P-glycoprotein substrate	No	Categorical (Yes/No)
	P-glycoprotein I inhibitor	No	Categorical (Yes/No)
	P-glycoprotein II inhibitor	No	Categorical (Yes/No)
Distribution	VDss (human)	-0.193	Numeric (log L/kg)
	Fraction unbound (human)	0.654	Numeric (Fu)
	BBB permeability	-0.907	Numeric (log BB)
	CNS permeability	-3.623	Numeric (log PS)
Metabolism	CYP2D6 substrate	No	Categorical (Yes/No)
	CYP3A4 substrate	No	Categorical (Yes/No)
	CYP1A2 inhibitior	No	Categorical (Yes/No)
	CYP2C19 inhibitior	No	Categorical (Yes/No)
	CYP2C9 inhibitior	No	Categorical (Yes/No)
	CYP2D6 inhibitior	No	Categorical (Yes/No)
	CYP3A4 inhibitior	No	Categorical (Yes/No)
Excretion	Total Clearance	1.672	Numeric (log ml/min/kg)
	Renal OCT2 substrate	No	Categorical (Yes/No)
Toxicity	AMES toxicity	No	Categorical (Yes/No)
	Max. tolerated dose (human)	1.395	Numeric (log mg/kg/day)
	hERG I inhibitor	No	Categorical (Yes/No)
	hERG II inhibitor	No	Categorical (Yes/No)
	Oral Rat Acute Toxicity (LD50)	1.834	Numeric (mol/kg)
	Oral Rat Chronic Toxicity (LOAEL)	3.109	Numeric (log mg/kg_bw/day)
	Hepatotoxicity	No	Categorical (Yes/No)
	Skin Sensitisation	No	Categorical (Yes/No)

**Abbreviations**: BBB, Blood-brain barrier, CNS, Central nervous system; CYP, Cytochrome; hERG, human ether-go-go gene; LD50- Lethal dose 50, OCT2, Organic cation transporter 2; VDss, volume of distribution.

**Table 4:** Binding energy calculation of ligand in 6CM4_Rosiridin and 7XN1_Rosiridin and non-bonded interaction energies from MMGBSA trajectories.

Energies (kcal/mol)*	**6CM4_Rosiridin**	**7XN1_Rosiridin**
ΔG_bind_	-46.71 ± 4.46	-29.22 ± 5.03
ΔG_bindCoulomb_	-12.89 ± 3.41	-11.2 ± 3.12
ΔG_bindCovalent_	5.1 ± 1.29	1.75 ± 0.89
ΔG_bindHbond_	-0.89 ± 0.18	-2.37 ± 0.27
ΔG_bind Lipo_	-23.06 ± 1.61	-15.45 ± 2.09
ΔG_bindSolvGB_	27.55 ± 2.43	33.07 ± 2.18
ΔG_bindvdW_	-42.51 ± 1.63	-35.03 ± 2.29

**Note**: *all the values presented in kcal/mol.

## Data Availability

Data supporting this study are included within the article and/or supporting materials.

## LIST OF ABBREVIATIONS

PD: Parkinson's disease
ROT: Rotenone
DA: Dopamine
NBT: Nitro Blue Tetrazolium
AChE: Acetylcholinesterase
PK: Pharmacokinetic
